# Predictors of intra-operative blood loss and blood transfusion in orthognathic surgery: a retrospective cohort study in 92 patients

**DOI:** 10.1186/s13037-014-0041-6

**Published:** 2014-10-02

**Authors:** Maisa O Al-Sebaei

**Affiliations:** Department of Oral and Maxillofacial Surgery, King AbdulAziz University, Faculty of Dentistry, PO Box 80209, Jeddah, 21589 Kingdom of Saudi Arabia

**Keywords:** Blood transfusion, Hypotensive anesthesia, Blood loss, Bimaxillary surgery, Orthognathic surgery, Hemoglobin

## Abstract

**Background:**

Patients undergoing orthognathic procedures can require blood transfusions. The objectives of this study were to evaluate the predictors of intra-operative blood loss in patients undergoing orthognathic procedures and the transfusion rates and practices of our institution.

**Materials and methods:**

This retrospective study included 92 patients who underwent the following four types of orthognathic procedures: Group 1, bimaxillary; Group 2, bimaxillary with bone grafts; Group 3, LeFort I osteotomies; and Group 4, LeFort I osteotomies with bone grafts. The intra-operative blood loss, operative time, age, gender and pre- and post-operative HGB and HCT were assessed.

**Results:**

The mean blood loss for all groups was 650 ± 397.8 mL, and there were differences in blood loss between the four groups (p = 0.211). The mean operative time was 5 hours and 32 minutes. There were no differences in intra-operative blood loss between the genders or the BMI categories. The operative time was moderately correlated with the intra-operative blood loss (p < 0.001, r =0.332). Eighteen of the 92 patients (19.5%) received blood transfusions. The mean intra-operative blood loss was higher among the patients who received transfusions (p < 0.001).

**Conclusions:**

The only predictor of intra-operative blood loss was operative time. The observed transfusion rate was higher than those that have been reported for similar procedures; thus, our institution needs to revisit our transfusion policy and use more time-efficient techniques in the operating room.

## Introduction

Orthognathic surgery for the correction of dento-facial deformities is a well-established procedure. Due to the high vascularity of the maxillofacial region, these procedures result in blood loss that can require allogeneic blood transfusions. There is an interest in the literature in the amount of blood loss during these procedures and the transfusion requirements of patients undergoing bimaxillary procedures. The length of the surgery, the experience of the surgeon, the gender of the patient [1] and type of procedure [2] are all factors that have reported to affect blood loss.

With the application of the many available techniques for minimizing intraoperative blood loss, the requirements of allogeneic blood transfusion have decreased in the past decade [3].

The use of good operative techniques, knowledge of the anatomy and the use of hypotensive anesthesia [4] are well-documented methods for reducing blood loss. The success of anti-fibrinolytic agents, such as tranexamic acid, in the reduction of intra-operative blood loss has also been reported [5].

The transfusion requirements for a healthy patient undergoing elective surgery do not depend on a single criterion. Although transfusion triggers have been defined in controlled trials as 7–8 g/dL [6], the decision to transfuse depends on the perioperative vital signs, hemoglobin concentration, patient co-morbidities and good clinical judgment.

The aims of my study were to evaluate the predictors of intra-operative blood loss and to assess the transfusion rate and practices related to orthognathic procedures in our institution of the King AbdulAziz University Hospital, Jeddah, Saudi Arabia.

## Materials and methods

This study was approved by the Unit of Biomedical Ethics Research Committee, Faculty of Medicine, King AbdulAziz University. A total of 100 consecutive patient records from 2009 to 2012 were retrospectively analyzed. Eight of these records were excluded due to incomplete data.

### Patients and procedures

A total of 92 patient records were included in the study. All of the patients underwent either a single-piece LeFort I osteotomy (LFI), a bilateral sagittal split osteotomy (BSSO) or a maxillary single-piece LFI bimaxillary orthognathic surgery with or without an autogenous free bone graft from a distant site (the anterior iliac crest or cranial bone graft) at King AbdulAziz University Hospital.

The patients were grouped into four categories:

1. bimaxillary surgery (LFI and BSSO), *n* = 42
2. bimaxillary surgery (LFI and BSSO) with a bone graft, *n* = 19
3. maxillary surgery (LFI), *n* = 21
4. maxillary surgery (LFI) with a bone graft, *n* = 10

Prior to the surgery, the American Society of Anesthesiologists status (ASA) was determined. Eighty-four of the 92 (87%) patients were classified as ASA I, and 8/92 (13%) were classified as ASA II patients. None of the patients had cardiovascular, cerebrovascular, or coagulation disorders or renal disease. All surgical procedures were performed by one of two consultant oral and maxillofacial surgeons with one or more trainees from the intermediate to senior level. The patients received 2 mg of midazolam pre-operatively in the holding area, followed by the induction of general anesthesia in the operating room using the standard anesthesia protocol in our institution (propofol induction, sevoflurane inhalation and muscle relaxant). An intravenous crystalloid solution was administered to the patients at 0.5-1.0 mL/kg per hour. The following hemodynamic parameters were used to assess intravascular volumes status: heart rate, arterial blood pressure, peripheral oxygen saturation, and urine output. Hypotensive anesthesia was performed during the maxillary component of the surgery. This anesthesia achieved by decreasing the blood pressure to a mean of 50–60 mmHg via an increase in the inhaled anesthetic.

The estimated intra-operative blood loss was recorded as per the protocol of our institution and was calculated by subtracting the amount of irrigation fluid used from the amount of blood visible in the suction collection canister.

### Data

The following data were collected from the patients’ records: age, gender, weight, height, body mass index (BMI), length of surgery, type of procedure, estimated intra-operative blood loss (EBL), and pre- and post-operative hemoglobin (HGB) and hematocrit (HCT). The pre-operative HGB and HCT were collected from the patient 1–2 days before the surgery, and the post-operative HGB and HCT were collected 6–24 hours after the surgery. The volume of intra-operative blood loss was also evaluated according to the total length of the surgery. The operative length was classified into the following three categories: 1–3 hours, 3–6 hours, and 6–10 hours. The individual cases that received packed red blood cell (pRBC) transfusions and the lowest intra-operative HGB values were recorded. The patients who did and did not receive transfusions were further classified according to the lowest intra-operative HGB for which a value of 7 g/dL was used as a “transfusion trigger”.

### Statistical analyses

The data were entered into a data entry sheet and descriptive statistics were used to present the demographics and the pre-operative HGB and HCT data for both the females and males.

The mean differences in age, BMI, intra-operative blood loss (EBL), operative time, and pre- and postoperative HGB and HCT were compared between the four surgical groups using one-way ANOVAs with assumed normal distributions. The relationship between operative time and intra-operative blood loss was assessed using a one-way ANOVA.

To evaluate whether intra-operative blood loss differed based on the type of procedure, gender or BMI category, one-way ANOVAs with assumed normal distributions were performed. The correlations of the intra-operative blood loss with the operative time, age and pre-operative HGB were assessed using Pearson product–moment correlation coefficients. A *t*-test was used to compare the EBLs of the patients who underwent blood transfusions and those of the patients who did not. A chi-square test was used to compare the utilization of blood transfusions between patients with HGB values above and below 7 g/dL.

All statistical analyses were performed using SPSS, version 20 (SPSS, Inc. IBM, Chicago, IL, USA). The level of significance was set at p < 0.05.

## Results

### Demographic data and descriptive statistics

There were a total of 92 patients who underwent orthognathic surgery for the correction of a dento-facial deformity. Fifty-six of the 92 patients were females (61%) whose mean age was 23.4 years, and 36 of the 92 patients were males (39%) whose mean age was 22.6 years. The mean age for both genders was 23 ± 6 years, and the mean weight was 58.5 kg ± 12. The predominant BMI category was normal (55/92 patients, 59.8%). The mean operative time was 5 hours and 53 minutes (the range was 1:22 to 9:25). A summary of these data is presented in Table 1.

**Table 1:** **Demographic data and descriptive statistics of the patients included in the study**

**Demographics**			**N**	**%**
*Gender*	Male		36	39.1
	Female		56	60.9
	Total		92	100.0
*BMI groups*	Underweight		17	18.5
	Normal		55	59.8
	Overweight or Obese		20	21.7
	Total		92	100.0
	**Min**	**Max**	**Mean**	**SD**
*Age*	14	43	23.12	6.14
*Height* (*cm*)	145	187	163.34	8.7
*Weight* (*kg*)	38	95	58.58	12.23
*BMI*	14.87	32.11	21.85	3.62
*Operative time* (*hrs*:*min*)	1:13	9:15	5:32	1:52

### Blood parameters

The mean pre-operative HGB for the females was 12.39 ± 1.7 g/dL, and this group exhibited a mean post-operative drop of 3.0 g/dL. For the males, the mean pre-operative HGB was 15.11 ± 1.2 g/dL, and the mean post-operative drop was 3.7 g/dL as shown in Table 2.

**Table 2:** **Mean pre**-**operative and post**-**operative hemoglobin and hematocrit by gender**

**Gender**			**N**	**Mean**	**SD**	**Mean diff**
Male	Hemoglobin	Pre	36	15.11	1.2	3.7
		Post	36	11.44	2.3	
	Hematocrit	Pre	36	44.26	3.7	10.2
		Post	36	34.01	6.5	
Female	Hemoglobin	Pre	56	12.39	1.7	3.0
		Post	56	9.39	1.7	
	Hematocrit	Pre	56	37.20	4.2	8.9
		Post	56	28.30	4.6	

### Blood loss

The mean intra-operative blood loss for all 92 patients was 650 ± 397.8 mL with a range of 100 mL to 2500 mL. The patients whose procedures lasted between 1–3 hours exhibited less intra-operative bleeding (*p* <0.001) compared to the patients whose procedures lasted 3–6 hours or 6–10 hours. These data are shown in Table 3.

**Table 3:** **Mean intra**-**operative blood loss and its relationship to operative time**

**Length of surgery**	**N**	**Mean**	**SD**	**P-** **value**
1-3 hrs.	15	316.67	206.73	
>3-6 hrs.	36	713.89	435.17	
>6-10 hrs.	41	715.85	359.94	0.001*
Total	92	650.00	397.80	

*Significant using One-*Way ANOVA at p* < *0.05*.

### Differences in the means between the surgical and procedure groups

Neither age nor BMI were significantly different between the four surgical groups (*p* = 0.120 and *p* = 0.705, respectively). There were no differences in intra-operative blood loss between the four surgical groups (*p* =0.211). The operative time was significantly (*p* < 0.001) shorter for group 3 (LeFort I without a graft) as shown in Table 4.

**Table 4:** **Mean differences between the surgical groups**, **where**: ***Group 1***: ***Bimaxillary Surgery***, ***Group 2***: ***Bimaxillary with bone graft***, ***Group 3***: ***Maxillary***, ***Group 4***: ***Maxillary with bone graft***

**Groups**	**N**	**Age**	**BMI**	**EBL**	**Duration**	**PreHGB**	**PreHCT**	**PostHGB**	**PostHCT**
Group 1	42	24.69 ± 6.2	21.70 ± 3.8	680.95 ± 313.12	5:58 ± 1:19	12.94 ± 2.0	38.51 ± 4.8	9.76 ± 2.1	29.42 ± 5.6
Group 2	19	22.79 ± 6.8	21.48 ± 3.0	744.74 ± 367	6:28 ± 1:07	14.14 ± 1.8	41.54 ± 4.8	9.82 ± 1.6	29.26 ± 4.2
Group 3	21	20.76 ± 5.3	22.61 ± 3.9	495.24 ± 517.91	3:25 ± 2:00*	13.64 ± 2.3	40.66 ± 6.7	11.25 ± 2.6	33.58 ± 7.7*
Group 4	10	22.10 ± 5.0	21.59 ± 3.8	665.00 ± 456.47	6:26 ± 1:11	13.97 ± 1.2	41.61 ± 3.5	10.53 ± 2.0	31.29 ± 5.4
Total	92	23.12 ± 6.1	21.85 ± 3.6	650.00 ± 397.8	5:32 ± 1:52	13.46 ± 2.0	39.96 ± 5.3	10.20 ± 2.2	30.54 ± 6.1
p-value		0.102	0.750	0.211	<0.001*	0.119	0.100	0.059	0.050*

Using one-way ANOVA test across the groups.

*Where BMI* = *body mass index*, *EBL* = *estimated intra*-*operative blood loss*, *HGB* = *hemoglobin*, *HCT* = *hematocrit*.

*Significant at p < *0.05*.

### Predictors of blood loss

There were no differences in intra-operative blood loss, age, or BMI category between the four surgical groups (*p* = 0.211, *p* = 0.062 and *p* = 0.930, respectively; Table 5).

**Table 5:** **Comparison of intra**-**operative blood loss relative to procedure type**, **gender and BMI**

**One-way anova**		**N**	**Mean EBL**	**SD**	**p-value**
**Procedure type**	Group 1	42	680.95	313.1	0.211
	Group 2	19	744.74	367	
	Group 3	21	495.24	517.9	
	Group 4	10	665.00	456.5	
**Gender**	Male	36	759.72	522.1	0.062
	Female	56	579.46	274.6	
**BMI**	Underweight	17	617.65	301	0.930
	Normal	55	654.55	421.2	
	Overweight or Obese	20	665.00	419.6	
	*Total*	92	650.00	397.8	

**Figure 1:**
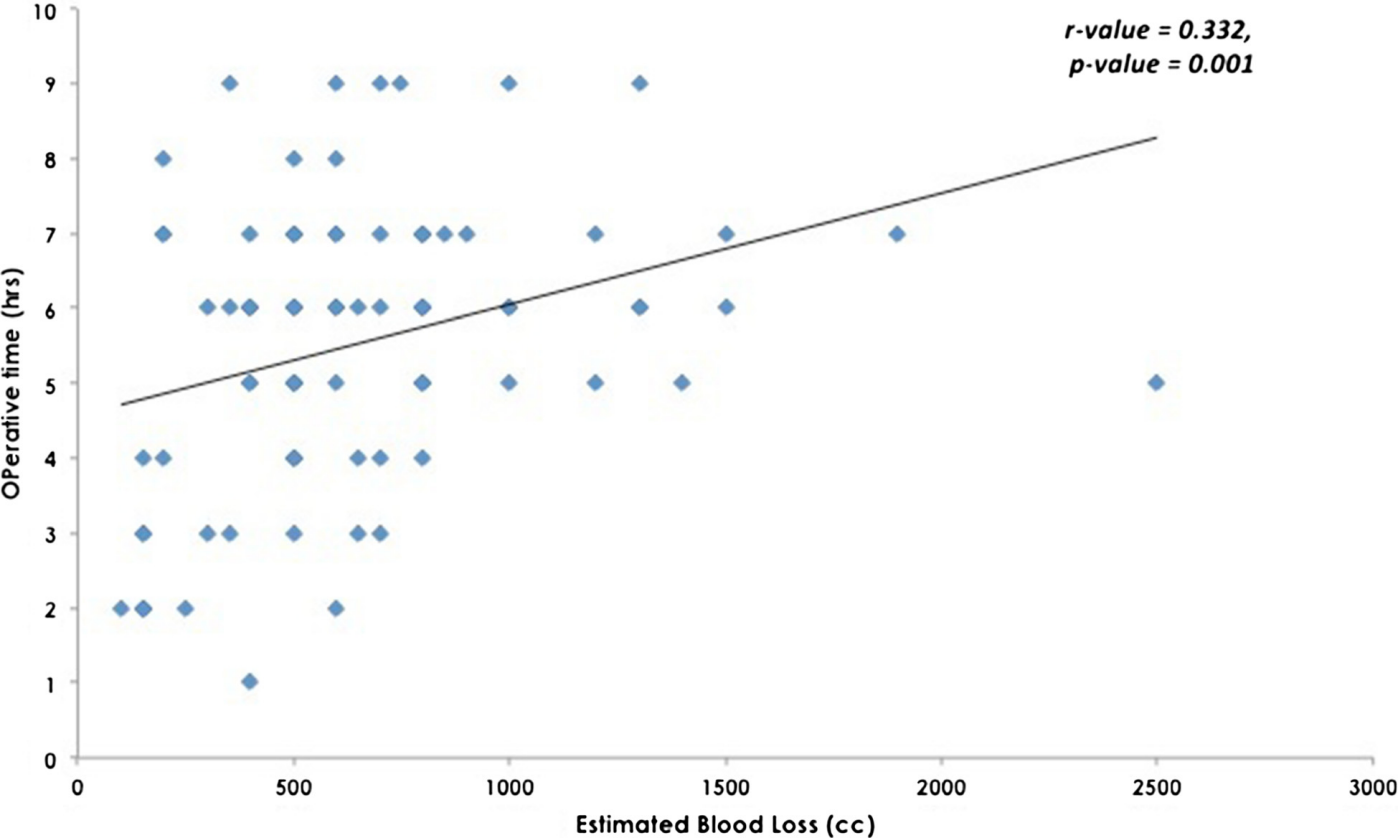
**A moderate correlation between intra-operative blood loss and operative time, using the Pearson Correlation Test, r- value was 0.33** **(** ***p*** **<** **0.001).**

There was a moderate positive correlation between intra-operative blood loss and operative time; the Pearson correlation value was 0.33 (*p* <0.001) as shown in Table 6 and Figure 1. Weak positive correlations were found between the age of the patient and the pre-operative HGB level and the intra-operative blood loss (r = 0.235 and 0.280, respectively).

**Table 6:** **Correlation of intra**-**operative blood loss to operative time**, **age and pre**-**operative hemoglobin level**

**Pearson correlation**		
**Operative time**	r	0.332
	p-value	0.001*
	N	92
**Age**	r	0.235
	p-value	0.024*
	N	92
**Pre**-**operative hemoglobin**	r	0.280
	p-value	0.007*
	N	92

*Significant using Pearson’*s Correlation Coefficient at p* < *0.05*.

### Packed Red Blood Cell Transfusion

Overall, 18 of the 92 (19.5%) patients received a blood transfusion. The mean EBL for the patients who received a transfusion was 975 ± 548 ml, which was significantly higher (p <0.001) than the mean EBL for those who did not receive a transfusion (570 ml ± 308 ml). The distributions of transfused patients for each of the surgical groups are shown in Table 7.

**Table 7:** **Mean intra**-**operative blood loss** (**EBL**) **for patients who underwent transfusion and those who did not**, **and the distribution among the surgical groups**

**Blood transfusion**		**N**	**Mean EBL**	**SD**	**P value**
Not transfused		74	570	308	<0.0001*
Transfused	Group 1	11	813.63	342.11	
	Group 2	4	875	340	
	Group 3	2	1600	1275	
	Group 4	1	1900	0	
	Total transfused	18	975	548	

*Significant using student’*s t*-*test at p* < *0.05*.

A total of 21 units of packed red blood cells (pRBC) were transfused, and one unit was used per patient with the exception of one male patient who received 3 units intra-operatively due to an active bleeding episode during the maxillary surgery.

**Figure 2:**
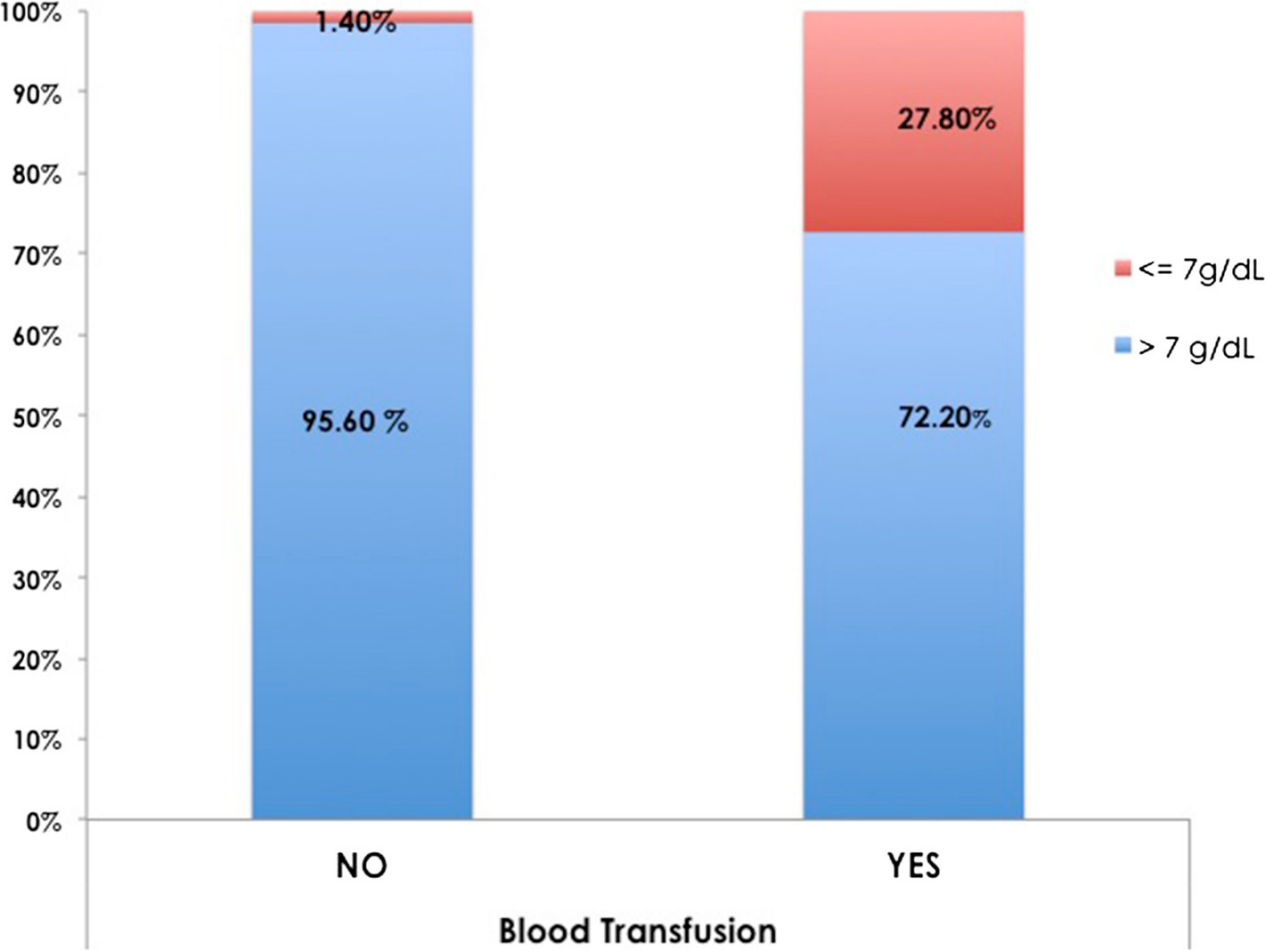
The number of patients who underwent blood transfusion compared to those who did not, using a HGB of 7 g/dL as a “transfusion trigger”. *Significant using Chi-*Square test at p* < *0.05*.

At the times at which the decisions for blood transfusion were made decision for the 18 patients who received transfusions, only 5 (27.8%) patients had reached the lowest HGB value of 7 g/dL or less, and 13/18 patients (72.2%) were above 7 g/dL. One patient with a HGB below than 7 g/dL was not transfused as shown in Figure 2.

Ten patients received intraoperative red blood cell transfusions with a total of 14 units. All patients received one unit each with the exception of one male patient who received 3 units due to excessive bleeding from the maxilla. The patient lost 2500 mL of blood, and his HGB declined to 5.5 g/dL. This was the only patient who exhibited class III hypovolemic shock, and he lost an estimated 40% of his total blood volume.

Eight patients received postoperative blood transfusions (24 to 48 hours after surgery), with a total of 8 units, and each of these patients received one unit. At the time of transfusion, these patients vital signs were consistent with class I hypovolemic shock.

### Cross-Match: Transfusion Ratio

The data were used to calculate the C: T ratio, which is defined as the number of cross-matched units used (perioperative and until hospital discharge) divided by the number of cross-matched units requested. The total of the cross-matched units 92, and the total number of units utilized was 18. The cross-match/transfusion ratio was 5.1:1.

## Discussion

Orthognathic procedures for the correction of dentofacial deformities are complex surgical procedures for which a considerable amount of blood loss is anticipated due to the vascular natures of the maxillary and mandibular structures. Pre-operative assessments should aim to evaluate ASA status and pre-operative HGB. The reductions in HGB that were observed in both the males and females in our study were greater than those that have been reported in other studies [2,7]. Perez reported that this reduction was not different between ASA I and ASA II patients who exhibited reductions of 1.62 and 1.71, respectively [2]. In contrast, Fenner et al. reported reductions of 3.4 among males and 3.2 among females, but the mean pre-operative HGBs of the females (13.4) and males (15.5) were higher [7].

The mean pre-operative HGB was within the normal range for the females but was close to the lower end of normal. According to the WHO classification, the cut-offs for anemia are 12 g/dL for females and 13 g/dL for males [8]. In Saudi Arabia, Alquaiz [9] found that 40% of women of child bearing age suffered from anemia in the city of Riyadh. These authors also found a mean HGB of 12.35 (±1.80) g/dL. This parameter should be taken considered when planning orthognathic procedures, particularly procedures for females in our society, by addressing low HGB levels with iron supplements and/or referrals to a specialist prior to surgery for optimization.

The study groups of the present study exhibited comparable ages and BMIs. Our results also indicated that the amounts of blood lost did not differ between the surgical sub-types (i.e., bimaxillary and maxillary and with or without a graft), which might indicate that the addition of another surgical site would not increase the blood loss significantly. However, the mean EBL was higher than those that have been reported in other studies [2,10,11]. The mean EBL during orthognathic procedures using hypotensive anesthesia varies in the literature, and only one study has reported a range between 50 and 5000 mL [10]. Intraoperative blood loss in orthognathic surgery was evaluated in a systematic review by Pineiro-Aguilar et al. [3]. These authors found a mean value of 436.11 ± 207.89 mL across a total of 17 studies that met the review criteria.

The predictors of intraoperative blood loss during orthognathic procedures also vary in the literature. Some authors have reported correlations with gender [1], operative time [1,12] and surgeon experience [1].

Consistent with our findings, some studies have found no significant predictors of EBL when examining factors such as gender, preoperative blood ingredients [11], segmentation of the maxilla, and Angle classification [13].

In general, the operative time did not vary with the type of surgery with the exception of the maxillary surgery without a graft (group 3) for which this time was significantly shorter.

The average operating time in this study (the operative time regardless of the addition of the bone graft procedure) was 5 hours and 32 minutes (332 minutes), which is higher than the previously reported times [7,11,14,15]. We also found a moderate correlation between EBL and the duration of the operation, which is also consistent with other studies [13,16].

The relationship between operative time and EBL is not a strong linear one; i.e., blood loss does not consistently increase over time. The majority of intra-operative blood loss is expected to occur in the beginning of the procedure during the performance of the osteotomies. Blood loss decreases as the multiple wounds are sutured. Hour-by-hour records of EBL were not available in the patients’ records.

The longer surgical time observed in this study could be explained by several factors. First, the complexity of the cases might have been responsible because patients present with severe dento-facial deformities primarily due to a lack of early intervention and access to healthcare and inter-family marriages. Second, our institution is a training facility for oral and maxillofacial surgery residents. A participating resident might be at an intermediate or senior level without much experience in orthognathic procedures, and time is required to allow the resident to perform certain parts of the procedure under direct supervision.

It is the policy of our institution that each patient provides two units of blood that can be donated by family or friends prior to surgery. The rationale behind this policy is to encourage the community to donate blood products to the blood bank. Generally, our community is not responsive to blood drives unless a relative is undergoing surgery.

It is also the policy of our hospital to cross-match one unit of blood for orthognathic surgeries. In our study, 92 units were cross-matched, and only 21 of these units were used. Thus, the match-to-transfusion ratio (C:T) was 5.1:1. According to the British Society of Hematology (BSH), blood should not be available for surgery if the usage is below 50%, which is equivalent to a C:T of 2:1 [17]. Any C:T above 2:1 indicates that units of blood need to be ordered and reserved for the patient and not utilized. In addition to the added cost that this limitation imposes on the blood bank, it renders some units unavailable for other patients who might need those units and decreases the shelf-life of those particular units. Our institution needs to revisit this cross-matching policy because it appears cross-matched units are being over-ordered.

The literature supports the notion that is there is no need to cross-match units of pRBC for orthognathic procedures [7,18,19] and that, even with reductions of HGB and HCT, the bodies of in a hemodynamically stable patients are able to compensate for the blood loss.

Many techniques to reduce blood loss in orthognathic surgery have been described. The most established and well documented of these techniques is hypotensive anesthesia [3,11,15].

In a prospective, double blind, randomized, controlled clinical study Ervens et al. found that hypotensive anesthesia significantly reduced blood loss and minimized transfusion requirements compared to isovolemic hemodilution [4].

Another widely used method is the peri-operative administration of tranexamic acid which has been proven to reduce intra-operative blood loss in bimaxillary procedures in randomized clinical trials [5,20-22].

Blood transfusion is not without risks; this procedure carries the risks of transfusion-transmissible infections [23], immunological transfusion reactions and mis-transfusions [24].

Autogenous blood is considered safer and to reduce the risk of transfusion complications [25]; however, many recent studies have not recommended the use of autologous blood donation as a routine part of orthognathic surgical procedures [7,12,18,19].

The reported transfusion rates for non-donor patients undergoing orthognathic procedures using hypotensive anesthesia over the last decade have been found to range from 0% to 8% [4,7,26,27]. Our data revealed a rate of blood transfusion (19.5%) that is higher than many of the rates that have been reported for similar procedures in the literature.

The criteria for transfusion for peri-operative patients have been the subject of debate and study [6,17]. The decision to transfuse patients undergoing orthognathic procedures should rely on the following factors: (*a*) the constant monitoring of blood loss with periodic assessment of the surgical field to identify ongoing or active bleeding; (*b*) monitoring of the intra-operative HGB (In controlled trials, transfusion triggers have been defined as 7–8 g/dL for healthy patients [6]);(*c*) monitoring for the presence of inadequate perfusion and oxygenation of the vital organs using dynamic parameters (i.e., blood pressure, heart rate, temperature, oxygen saturation and urine output; tachycardia, lactic acidosis, and increased total body oxygen extraction (SvO2 < 65%) are considered to be “physiologic transfusion triggers” provided that normovolemia and the proper depth of anesthesia are maintained [28,29]); and (*d*) good communication with the surgical team.

The intravascular status and any evidence of hypovolemia are evaluated during orthognathic surgeries using hemodynamic parameters for which changes over time are more significant than the actual measured levels. Additionally, the use of an arterial line is a common practice in our hospital during orthognathic cases; therefore, the anesthesiologist has access to the intraoperative blood PH, arterial oxygen concentration and an accurate HGB level.

The patients in our study were young, ASA I and ASA II and had no histories of cardiac disease. These patients had the physiologic reserves to compensate for the blood loss provided that their HGB level was 7–8 and that they were asymptomatic [6]. Few isolated cases of orthognathic procedures are considered to be life-threatening [30], and many of these have pre-existing coagulopathies that justify the ordering of a cross-match or auto-donation [12,31].

The following questions remains. Is our institution providing transfusions because they are readily available and cross-matched? And does the hemodynamic status of the patient justify the transfusion?

From a clinical standpoint and to minimize blood transfusions in this patient population, the patients’ preoperative HGBs need to be optimized prior to the orthognathic procedures. The vast majority of these procedures are elective and mean reductions of 3.0 g/dL and 3.7 g/dL for the females and males, respectively, were observed in our study. For females and males, minimums of 12 g/dL and 13 g/dL, respectively, should be set as preoperative minimums for orthognathic procedures. A referral to a specialist for correction of anemia followed by subsequent testing should precede the surgery. Furthermore, the use of adequate hypotensive anesthesia during the osteotomy phase of the procedure with a mean arterial blood pressure 30% below normal (50–60 mmHg) has been proven to be valuable in reducing intra-operative blood loss.

## Conclusions

The average intra-operative blood loss in our orthognathic cases was higher than those that have been reported for orthognathic cases and was moderately correlated with operative time. The transfusion rate was high at 19.5%, and 72.2% of those transfused patients received blood with an HGB above 7 g/dL. Intra-operative blood loss was not found to be correlated with the type of the procedure (i.e., maxillary or bimaxillary and with or without a bone graft), BMI or gender.

With the exception of controlled arterial hypotension, our institution does not follow any other procedures to decrease intraoperative blood loss. Techniques such as pre-operative autogenous blood donation and use of tranexamic acid should be considered to minimize the rate and risks of allogeneic blood transfusion. Furthermore, our hospital needs stricter transfusion criteria that are based on the patient’s maximum allowable blood loss, preoperative HGB and physiological reserve to avoid inappropriate transfusion practices.
